# Calcium mimics the chemotactic effect of conditioned media and is an effective inducer of bone regeneration

**DOI:** 10.1371/journal.pone.0210301

**Published:** 2019-01-04

**Authors:** Rubén Aquino-Martínez, David G. Monroe, Francesc Ventura

**Affiliations:** 1 Departament de Ciències Fisiològiques, Universitat de Barcelona, IDIBELL, L’Hospitalet de Llobregat, Barcelona, Spain; 2 Department of Medicine, Division of Endocrinology, Mayo Clinic College of Medicine, Rochester, MN, United States of America; Università degli Studi della Campania, ITALY

## Abstract

**Background:**

After bone resorption, ions and degraded organic components are co-released into the extracellular space. Ions and growth factors, although different in their biological nature, induce a common and coordinated chemotactic effect. Conditioned media has been used successfully in bone regeneration by promoting endogenous cell recruitment. Likewise, calcium alone act as a paracrine chemotactic signal, inducing the host’s undifferentiated progenitor cell infiltration into the implanted biomaterials. The aim of the present study was to compare the chemotactic effect of calcium and conditioned media in primary calvarial cells.

**Methods:**

The chemotactic cell response was evaluated *in vitro* using an agarose spot and a wound healing assay. In addition, we used a calvarial bone explant model *ex-vivo*. The healing potential was also tested through an *in vivo* model, a critical-size calvarial bone defect in mice. For the *in vivo* experiment, cell-free calcium-containing or conditioned media-containing scaffolds were implanted, and MSC’s seeded scaffolds were used as positive control. After seven weeks post-implantation, samples were retrieved, and bone regeneration was evaluated by μCT and histological analysis. Osteogenic gene expression was evaluated by qPCR.

**Results:**

We found that chemotactic cell migration in response to either calcium or conditioned media was equivalent *in vitro* and *ex vivo*. Accordingly, μCT analysis showed that bone regeneration induced by the MSC’s seeded scaffolds was similar to that obtained with cell-free calcium or conditioned media-containing scaffolds. Pre-treatment with SB202190, a highly selective p38 inhibitor, abrogated the chemotactic effect induced by conditioned media. In contrast, p38 activity was not essential for the calcium-induced chemotaxis. Moreover, BAPTA-AM treatment, a cytosolic calcium chelator, decreased the chemotactic effect and the expression of key osteogenic genes induced by calcium or conditioned media.

**Conclusion:**

We show that calcium ions alone not only mimic the conditioned media chemotactic effect, but also induce an osteogenic effect similar to that produced by transplanted MSC’s *in vivo*. Furthermore, the chemotactic effect induced by conditioned media is calcium and p38 dependent. The rise in cytosolic calcium might integrate the different signaling pathways triggered by conditioned media and extracellular Ca^2+^. This calcium-driven in situ bone regeneration is a promising and convenient alternative to promote endogenous cell recruitment into the injured bone site. This pre-clinical cell-free and growth factor-free approach might avoid the disadvantages of the *ex vivo* cell manipulation.

## Background

The regeneration of oral and maxillofacial bone defects is one of the most challenging procedures in the clinical setting [1]. Although bone is the hardest tissue in the human body, it can be incompletely formed congenitally, as in the case of cleft palate, or injured after trauma. When extensive bone damaged is produced, autografts or bone substitutes are required to restore anatomically and functionally such defects. Cell-based tissue engineering approaches have emerged as a promising alternative for autologous bone harvesting, but they require an appropriate donor site as cell source [2][3]. Therefore, an attractive strategy for bone regeneration is to identify effective chemotactic stimuli to recruit endogenous MSC’s into the injured bone, avoiding the *ex vivo* cell manipulation [4][5].

The beneficial effects of MSC’s transplantation and cell-based tissue engineering constructs rely on two primary mechanisms. First, they contribute to bone formation by their ability to differentiate into osteoblasts, although the survival rate of the implanted cells is low [6][7]. On the other hand, MSC’s also secrete multiple paracrine signaling molecules that recruits host mesenchymal progenitor cells [8] [9]. Increasing evidence suggests that this paracrine effect is the predominant osteogenic mechanism, reaching in some cases up to 80% of cell transplantation beneficial effects [6][10][11]. Since these paracrine signals are released and can be collected from the conditioned media during MSC’s culture, conditioned media has been used as a cell-free approach for bone regeneration [9]. Of note, MSC’s conditioned media produces an osteogenic effect comparable or stronger than transplanted cells [10][9]. Recently, it has also been reported that a specific mixture of cytokines, including IGF, VEGF and TGFβ1, can mimic the effect of the conditioned media for bone regeneration [12]. Therefore, bioactive molecules in conditioned media can be used as a cell-free approach, with equivalent effects than MSC’s transplantation.

During the sequence of bone formation and regeneration undifferentiated progenitor cells are attracted to specific sites by chemotactic signals, and gradually differentiate into bone forming osteoblasts[13][14]. These osteoprogenitor cells secrete a myriad of growth factors that are stored in a collagenous extracellular matrix, which eventually mineralizes [15]. Concentrations of soluble calcium in the bone microenvironment are in the mM range, [16][17] whereas the organic fraction containing the growth factors are present in a pico-nM range [18][19]. Among these stored growth factors in bone matrix are BMP2, TGFβ, PDGF, IGF, FGF, or PDGF [15] [20][21][22]. After bone resorption a mixture of dissolved ions and degraded organic components are released into the extracellular space. Despite inorganic ions and growth factors are different in their biological nature, they induce a common chemotactic effect on undifferentiated mesenchymal cells.

Recently, we reported that specific CaSO_4_ concentrations promote MSC’s recruitment and infiltration into a cell-free tissue engineering construct [23]. This chemotactic effect is calcium-dependent, since extracellular calcium chelation inhibits such effects [23]. Furthermore, Calcium Sensing Receptor (CaSR) inhibition also disrupted the MSC’s chemotactic response to calcium, showing that this receptor is also essential to induce cell recruitment [24]. In fact, extracellular calcium alone shows a cell migration effect, which is comparable to that induced by BMP-2 or VEGF [23][24].

Since both conditioned media and calcium ions induce bone regeneration by recruiting host’s MSC’s, we hypothesized that both conditions could have a similar paracrine chemotactic effect on calvarial cells. To demonstrate our hypothesis, we compare the chemotactic effects *in vitro*, and using a calvarial explant model *ex vivo*. Moreover, an *in vivo* calvarial bone defect model was used to compare their bone regeneration ability. We also evaluated the potential molecular mechanisms involved in this chemotactic effect. Our results may provide support for an alternative approach for bone regeneration that eliminates cell culture and transplantation, and does not require the use of recombinant growth factors.

## Methods

### Isolation and culture of Mesenchymal Stem Cells (MSC’s)

MSC’s were isolated following a previously described protocol [25]. Briefly, hind limbs from euthanized 6–8 weeks old Balb/C mice were aseptically dissected, and soft tissues were carefully removed. Both metaphyses were cut, and bone marrow was collected by inserting a 27-gauge needle and flushing DMEM containing 1% penicillin/streptomycin, 1mM pyruvate, 2mM glutamine and FBS 10%. Cells were filtered using a 70 μm strainer (BD, Falcon), seeded into T-75 flasks, and incubated at 37^o^ C. Non-adherent cells were discarded by changing media. When cells reached 70–80 of confluency they were washed twice with PBS, and treated with trypsin for 3 minutes at room temperature. Lifted cells were cultured and expanded to collect the conditioned media, and for the in *vivo* experiment.

### Calvarial cell isolation

Primary mouse calvarial osteoblasts were isolated following a protocol previously described [26]. Cells were obtained by sequential enzymatic digestion using Collagenase type 2 for 10 minutes at 37^o^ C. Collected cells were maintained in α-MEM supplemented with antibiotic/antimycotic (ThermoFisher Scientific), Glutamax and FBS 10% (GE Healthcare Life sciences HyClone laboratories). For osteoblast differentiation, confluent cells were cultured with supplemented α-MEM or supplemented α-MEM containing 5 mM CaCl_2_, and allowed to differentiate for 0, 3, 7 and 10 days. SB431542, a selective TGFB type I receptor inhibitor (Tocris), was solubilized using DMSO at a 10 mM stock concentration, and a final concentration of 10 μM. AG-1296, a selective PDGF receptor inhibitor (Abcam), was used at a final 10 μM concentration.

### Conditioned media preparation

Bone marrow MSC’s were seeded in T75 flasks with DMEM supplemented with 15% FBS. For the serum-free, cells at 70–80% of confluency were washed three times with PBS, and then serum free media containing 1% penicillin/streptomycin was used. Cells were incubated at 37^o^ C for 48 hours. The medium was collected and centrifuged at 10,000 rpm for 10 minutes to eliminate cell debris. Conditioned media was stored at 4^o^ C for subsequent experiments.

### Agarose spot assay

For the *in vitro* chemotaxis evaluation, we prepared an agarose spot assay, following a protocol previously described [27][28][29]. Low melting agarose (Sigma-Aldrich) was mixed with PBS to obtain a 1% concentration. The solution was heated until boiling and then cooled to 37^o^ C. In microcentrifuge tubes containing 50 μl of melted 1% agarose, we added 50 μl of PBS, conditioned medium or 10 mM CaSO_4_. Three separated 1μl spots were applied onto a 35 mm culture dish; one containing agarose/PBS, one agarose/conditioned media and one agarose/calcium. The spots were cooled at 4^o^ C for five minutes to allow solidification. Meanwhile, calvarial cells were treated with trypsin for 3 minutes. Media containing 10% FBS was added, and centrifuged at 1500 rpm for 10 minutes. Then, fresh media containing 1% FBS was used to prepare the cell suspension. This cell suspension was added into the dishes containing the three spots. After 24 hours the cells were stained with crystal violet for 20–30 minutes at room temperature. Migrated cells under each spot were counted using the ImageJ software. At least five different experiments were performed.

### Wound healing assay

Calvarial cells were seeded and grown to confluence. Twenty-four hours before the assay, media containing 1% FBS was used to culture the cells. The monolayer of confluent cells was scratched (wounded) using a 200 μl pipette tip, and the cells washed twice with PBS to eliminate detached cells and debris. PBS was replaced by serum free media (negative control), media with 10% FBS (positive control), conditioned media or 5 mM CaCl_2_. Cells were pre-treated for 1 hour with BAPTA-AM at a 10 μM concentration (ThermoFisher Scientific), or SB202190 at a 10 μM concentration (a selective p38 inhibitor, Sigma-Aldrich). Images were taken immediately after the scratch to measure the initial wounded area and the cells incubated at 37^o^. The cells were allowed to migrate for 24 hours and then fixed with PFA 4%. Image analyses was performed using ImageJ and normalized to the respective control.

### RT-qPCR

RNA was isolated using QIAzol (Qiagen), according to the manufacturer’s instructions. RNeasy Micro Kit and the QIAcube instrument (Qiagen) were also used following the manufacturer’s protocol. RNA quantification was performed by spectrophotometric analysis (Nanodrop, Thermo Scientific, USA). One μg of total RNA was reverse- transcribed using a High capacity cDNA kit (Applied Biosystems). Five ng of cDNA were used for reaction, and three replicates for each condition. ABI Prism Real-Time system instrument (Applied Biosystems) and SYBR Green reagent (Qiagen) were used for the qPCR analysis. The primer sequences are showed in S1 Table. Data was normalized to *Actb* (β-actin) expression, and median CT values were used for 2−ΔΔCT quantification.

### Western blot assay

Protein extracts from treated cells were prepared by using lysis buffer and protease inhibitors, as described previously [30]. Protein samples were subjected to SDS-PAGE and immunoblotting. Membranes were incubated with antibodies for Runx2 (Abcam, ab23981), Osterix (Thermo Fisher Scientific, PA5-40411) and GAPDH (Cell Signaling, 14C10) diluted at 1:1000. The bands were visualized using an ECL kit (LI-COR Odyssey System, LI-COR Biosciences).

### Immunofluorescence

Primary calvarial cells were seeded on 8 well chamber slide (Sigma-Aldrich) and treated with the different conditions for 48 hours. Cells in each group were fixed in 4% PFA for 15 minutes and permeabilized with 0.1% Triton X-100. After blocking with 10% goat serum for 1 hour at room temperature, the cells were incubated with Osterix antibody (1:500) for 1 hour. Then, they were incubated with the secondary antibody (goat α-rabbit Alexa 447) for 1 hour. DAPI Fluoromount (SouthernBiotech) was used as mounting medium and nuclear staining. Labeling was detected by using a laser scanning confocal microscope.

### Scaffold preparation

For the *in vivo* calvarial defect model, scaffolds were prepared as previously described [23]. Under sterile conditions, gelatin sponges (Gelita B. Braun) were cut into fragments. Independent 60 mm dishes containing 3 ml of serum free media, conditioned media or 20 mM of Ca^2+^ were used to soak the prepared gelatin fragments. They were stored overnight in the incubator at 37^o^. For the scaffolds containing cells, 7 x 10^5^ bone marrow MSC’s were seeded to each scaffold. To obtain an efficient cell attachment, the gelatin scaffold was pre-soaked in media, transferred into a 1.5 ml microcentrifuge tube containing the cell solution, and incubated in vertical position for 4–6 hours at 37^o^. Next, the seeded scaffolds were transferred into a 60 mm uncoated dish, 3 ml of supplemented media were added, and incubated overnight. Before the surgical procedure, 3 ml of melted 1% low melting agarose (37^o^ C) were added to each 60 mm dishes containing the seeded scaffolds, those soaked in serum free, conditioned media or calcium. The dishes were cooled for 5 minutes at 4^o^ C. Each gelatin scaffold was trimmed using a scalpel and implanted into the calvarial bone defect.

### *Ex vivo* calvarial explant culture model

To evaluate the recruitment and healing potential of calcium ions and conditioned media, we performed an *ex vivo* explant culture model, as described previously [31][32]. Briefly, calvarie were dissected and parietal bones were split following the sagittal suture using surgical scissors. Full circular defects of 0.8 mm in diameter were created in each parietal bone using a stainless-steel needle. Calvarie from 6 months old mice were prepared to minimize spontaneous healing. Gelatin sponges, cut in 3x3x3 mm fragments, were soaked in the conditioned media or calcium-containing solution. Serum free media was used as negative control, and DMEM 15% FBS with or without 2.5 mM CaSO_4_ was used for the *ex vivo* model. Soaked scaffolds were inserted into the defect and each condition was cultured separately in a 24 well plate. Calvaria were cultured at 37^o^ for 2 weeks and the respective media was refreshed every 3 days.

### *In vivo* critical size calvarial defect model

The surgical protocol used to create a full thickness circular bone defect in the parietal bone of mice was described previously [33][34]. Ten-week-old Balb/C mice were used for this study. They were anesthetized by isoflurane inhalation, and buprenorphine was administrated for intraoperative analgesia. To expose the parietal bone, a longitudinal incision was performed, and periosteum conserved. The circular defect was created by using a 5 mm outer diameter trephine and a dental implant motor. The bone defect was covered with a scaffold according to the corresponding condition of study. Seven weeks after the scaffold implantation the mice were euthanized by CO_2_ inhalation, the heads were fixed in PFA 4%, and stored at 4^o^ C. All the animal procedures were performed in accordance with the protocol approved by the Ethics Committee for Animal Experimentation of the University of Barcelona and by the Generalitat of Catalunya.

### Experimental groups for the *in vivo* study

Thirty-two mice were divided in four groups, and the defects covered as follow: Group 1: agarose-gelatin scaffold (SCAFFOLD). Group 2: SCAFFOLD seeded with MSC’s. Group 3: SCAFFOLD soaked in CaSO_4_ 20mM. Group 4: SCAFFOLD soaked in conditioned media.

### Micro CT analysis

The scanning was performed with Skyscan 1076 (Skyscan, Kontich, Belgium), with the following parameters: 49 kv, 200 μA, exposure time 500 ms, 1 mm aluminum filter and 180^o^ rotation. Data reconstruction and three-dimensional models were made with NRecon and CTAn software, respectively.

### Histological analysis

After scanning, the calvariae were decalcified for 48 hours using Decalcifier II (Leica). Then, the samples were put in 30% sucrose solution overnight. The next day the samples were washed with PBS, embedded in OCT and snap-frozen in liquid nitrogen. Samples were stored at -80^o^ C for future histological evaluations. Slides of 6 μm were stained with hematoxilyn/eosin, or Masson’s Trichrome.

### Statistical analysis

Data is shown as mean ± SEM. The analyses were performed using Student’s t and the GraphPad software. The differences were considered significant at p < 0.05 * or #, p < 0.01** or ##, and p < 0.001*** or ###.

## Results

### 3.1 Chemotactic effect of calcium and conditioned media on primary calvarial cells

**Fig 1:**
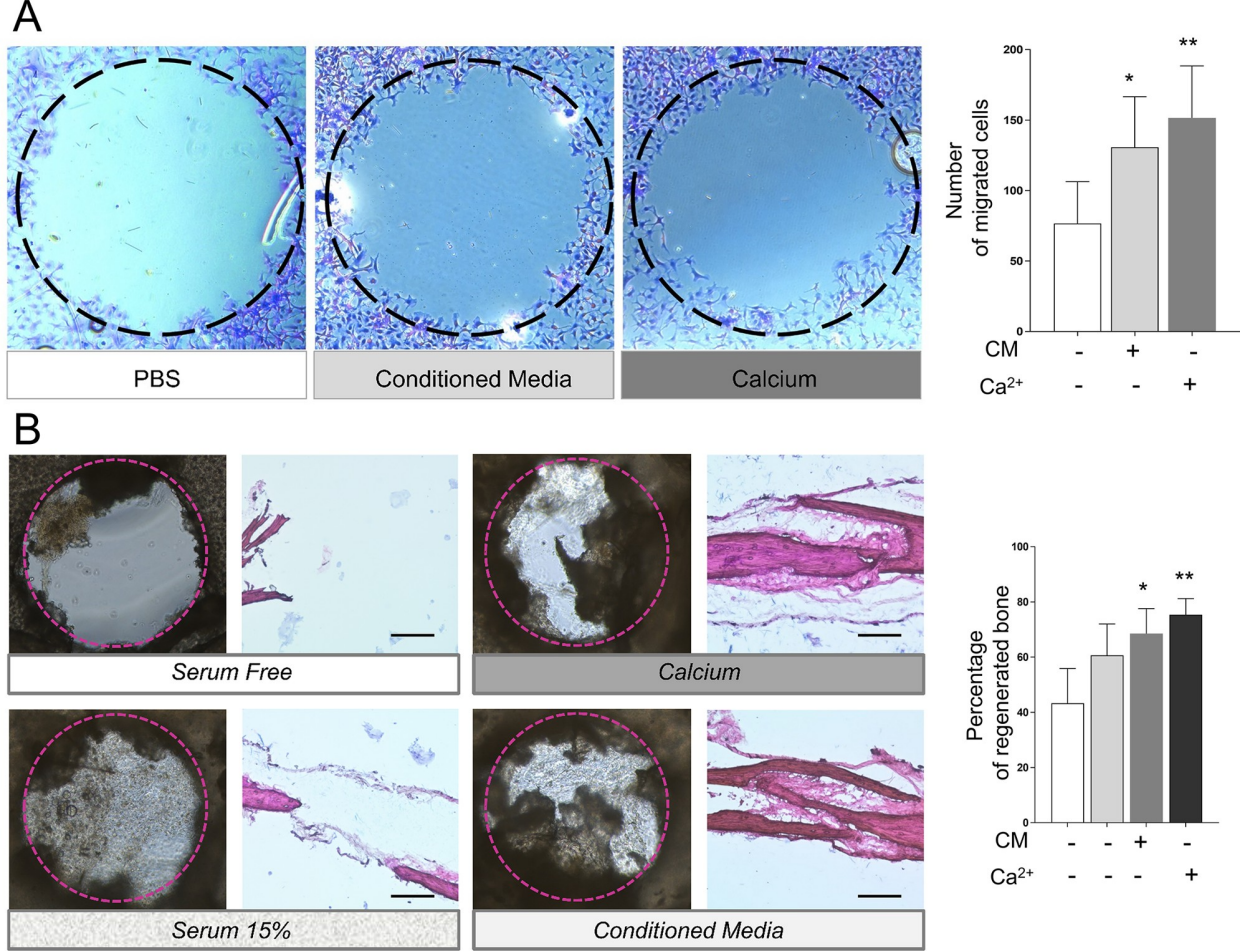
The effect of calcium or conditioned media on migration was evaluated by an agarose spot assay. (A) Three spots were plated on the same dish, one containing PBS, 10 mM CaSO_4_ or conditioned media. Primary calvarial cell migration under each agarose spot was evaluated and quantified using ImageJ software. The experiment was repeated five times. (B) Calvaria were divided into two halves and a full thickness defect of 0.8 mm of diameter was created. Halves were placed individually in a 24-well plate serum free media (negative control), 15% FBS, 2.5 mM of CaSO_4_ in 15% FBS media or conditioned media (n = 10). After two weeks, calvaria were fixed, images taken, and then prepared for histological evaluation. The percentage of regenerated bone was quantified by ImageJ. Representative images of each group are displayed. 20X scale = 400 μm. Differences are considered significant at p<0.05.

We analyzed the chemotactic ability of Ca^2+^ and conditioned media by using an agarose spot assay. This assay allows to measure different chemotactic signals under uniform cell culture conditions [29]. Cell migration in response to both stimuli was higher compared to the control condition (Fig 1A). Because of the limitations of two-dimensional cell culture, we decided to use an additional model that closely resembles the calvarial bone environment. An *ex vivo* calvarial explant model was used to compare the recruitment ability and healing potential of Ca^2+^and conditioned media [31][32] (Fig 1B). In agreement with the *in vitro* results, both chemoattractants generated a greater bone-like tissue formation compared to the control explants, cultured with serum free media. Indeed, the percentage of regenerated bone observed in the calcium treated conditions and those treated with conditioned media was higher compared to control. Interestingly, most of the scaffolds cultured with serum free media shrank or tore over time, as shown in Fig 1B (left upper panel). Histological samples stained with hematoxylin and eosin showed that those explants cultured with calcium enriched media or conditioned media were able to promote new bone-like tissue formation Our results using an *in vitro* and an *ex vivo* calvarial explant model suggest that extracellular calcium is as effective as conditioned media inducing the recruitment of calvarial cells.

### 3.2 Osteogenic effect of calcium, conditioned media and transplanted MSC’s in a calvarial defect model *in vivo*

**Fig 2:**
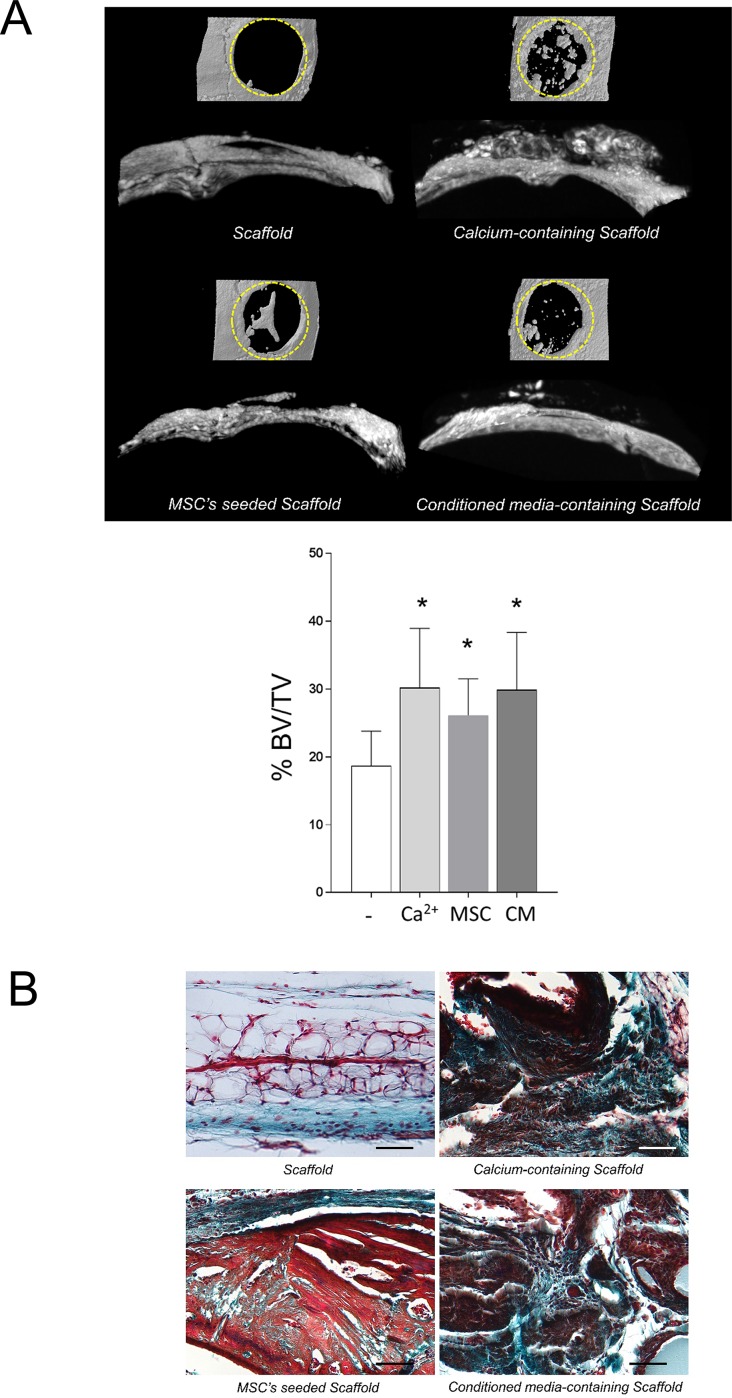
(A) A critical size calvarial bone defect of 5 mm in diameter was created in the parietal bone of mice (n = 8). After seven weeks, the implanted scaffolds were collected, fixed and prepared for microCT and histological analysis. Representative images of the control scaffold soaked in PBS, MSC’s seeded scaffold, cell-free 20 mM CaSO_4_ or conditioned media-soaked scaffolds are shown. Quantitative analysis is presented by bone volume/tissue volume (BV/TV %) (B) Masson’s trichrome staining shows the infiltration of host’s cells into the implanted cell-free scaffolds. 20X scale = 400 μm. Differences are considered significant at p<0.05.

To extend our results, we also evaluated the osteogenic potential of Ca^2+^ and conditioned media from MSC’s in an *in vivo* context. A bone defect of 5 mm in diameter was created in the parietal bone of mice, and covered with a scaffold alone, a scaffold seeded with MSC’s, a scaffold containing Ca^2+^ or conditioned media. To generate a more challenging calvarial defect, we used a minimal irrigation during the surgical procedure [34]. After seven weeks the calvariae were collected and analyzed by μCT to evaluate the new bone formation. The analysis of the reconstructed 3D images presented as percentage of the regenerated area (BV/TV) showed that a scarce bone was regenerated in the control group (Fig 2A). A comparable bone regeneration was observed among the different osteoinductive conditions. On the other hand, decalcified sections stained with Masson’s Trichrome were used to evaluate the morphological features of the regenerated bone. The histological evaluation showed the presence of scattered host cells and a poor bone formation rate in the control group (Fig 2B). In contrast, cluster of recruited cells and bone-like structures were observed in both the scaffolds with Ca^2+^ and conditioned media. Since cell-free scaffolds containing calcium or conditioned media were implanted, these results suggest that both chemoattractants induced a comparable host’s cell recruitment. Furthermore, the bone regeneration promoted by the scaffolds seeded with MSC’s was equivalent to that induced by calcium or conditioned media containing scaffolds.

### 3.3 The mRNA pattern expression of endogenous growth factors is up-regulated by calcium *in vitro*

**Fig 3:**
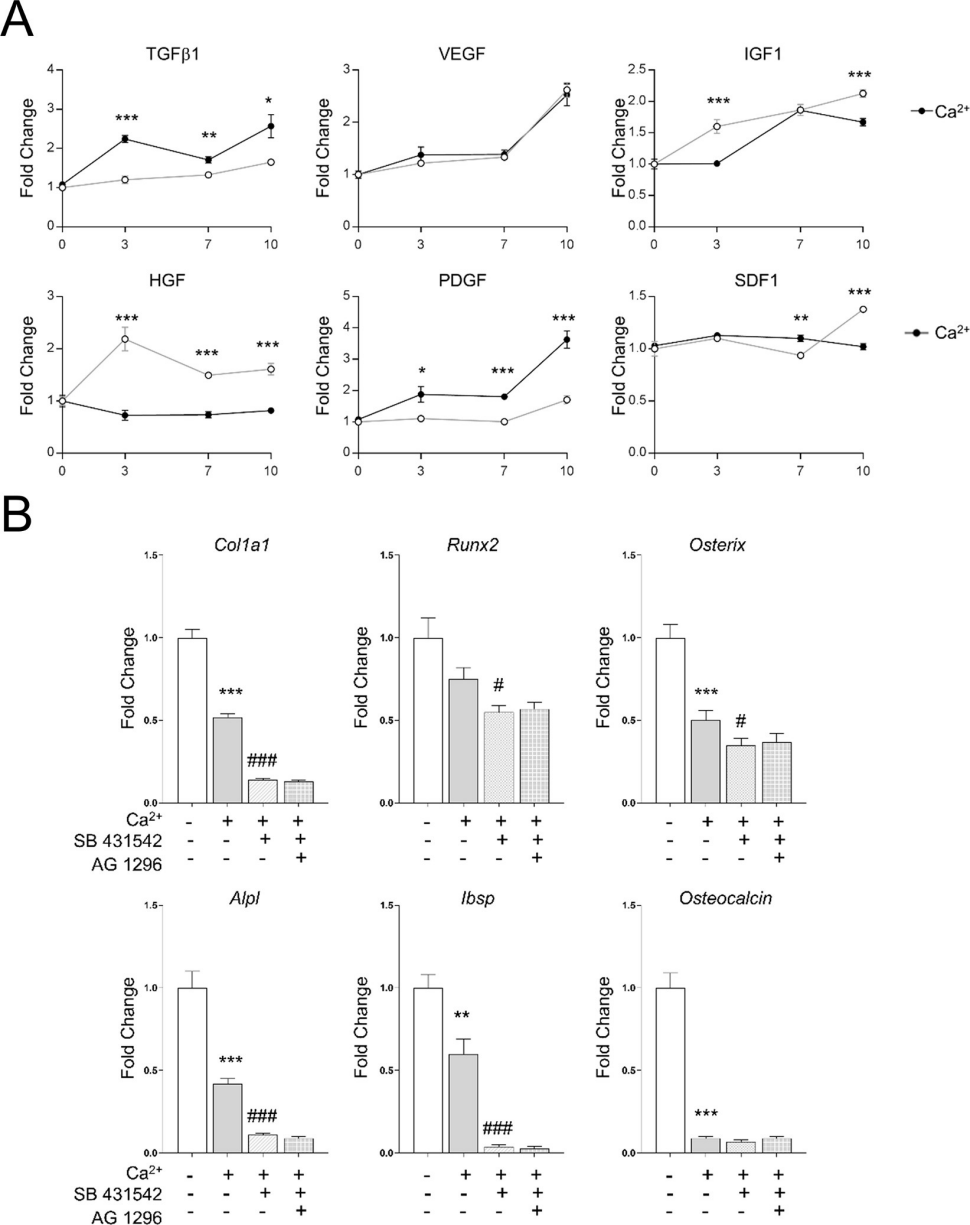
(A). Primary calvarial cells were cultured with supplemented DMEM or additional 5mM calcium for 3, 7 and 10 days. The mRNA expression was evaluated by qPCR. The effect of calcium (black circles) and media alone (empty circles) on primary calvarial cells is displayed. (B) Expression of osteogenic genes treated for 48 h with 5 mM calcium, SB431542 and AG-1296 (10 μM), a selective TGFβ1 and PDGF receptor inhibitor respectively. Data is shown as mean ± SEM. Differences considered significant at *p< 0.05, **p < 0.01, and ***p < 0.001 respect to control, and # p< 0.05, ## p < 0.01, and ### p < 0.001 respect to calcium treated cells.

To investigate the effect of Ca^2+^ on the expression of some key chemotactic factors, calvarial cells were cultured in supplemented DMEM alone, or containing CaCl_2_ 5mM for 0, 3, 7 and 10 days. Since it has been reported that IGF1, VEGF, TGFβ1 and HGF[12][35][36], are some of the critical factors to enhance MSC’s migration, we evaluated their mRNA pattern expression induced by calcium. The qPCR analysis showed that the addition of Ca^2+^ 5mM increased the expression of TGFβ1 and PDGF, and abrogated the increase of the HGF expression. (Fig 3A). We wanted to analyze the role of TGFβ signaling in the calcium-induced differentiation of the bone cells. Calvarial cells were pre-treated with SB431542, a selective TGFβ receptor inhibitor. After 7 days, the *Col1a1*, *Runx2*, *Osterix*, *Alpl* and *Ibsp* mRNA expression was significantly down-regulated compared to the control (Fig 3B). Additionally, we also analyzed the effect of AG-1296, a selective PDGF receptor inhibitor. We found that no additional effects were induced by the PDGF inhibitor. These results suggest that the *Tgfb1* mRNA expression might play a relevant role modulating the early osteogenic effects induced by calcium on osteoprogenitors cells.

### 3.4 P38 activity is differentially required for calcium and conditioned media induced chemotaxis

**Fig 4:**
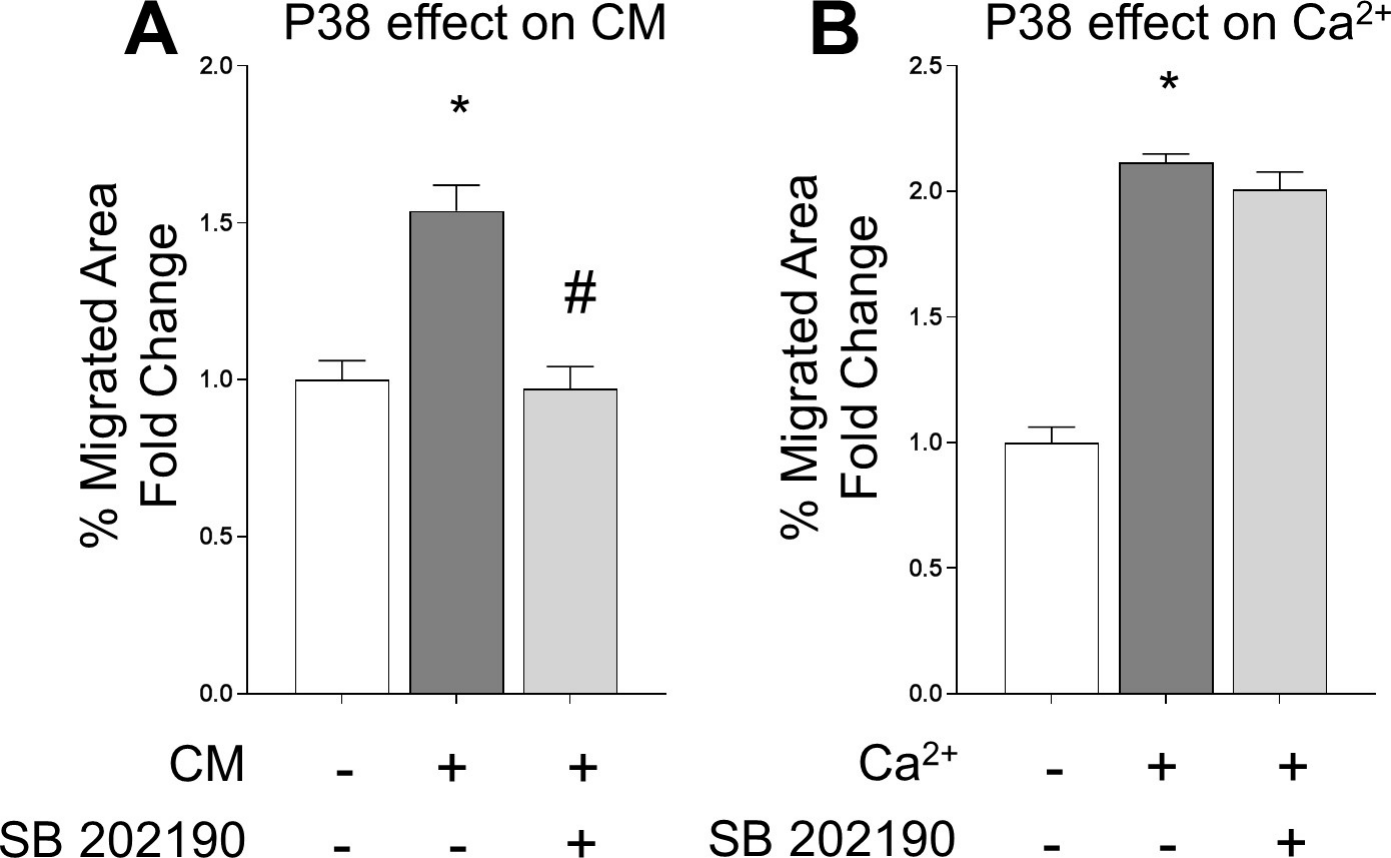
(A) A wound healing assay was used to test the role of p38 activity in the calcium and conditioned media induced chemotaxis. Cells were treated with SB202190 (10 μM), a highly selective p38 inhibitor, the cell monolayer was scratched and cell migration was quantified after 24 hours. Data is presented as mean ± SEM. Differences considered significant at *p< 0.05, respect to control, and #p < 0.05 respect to calcium-treated cells.

Given the critical role of p38 in cytoskeletal actin polymerization and cell migration [37][38], we also evaluated the involvement of p38 activity on cell migration elicited by extracellular calcium and conditioned media. We found that calvarial cells incubated with SB201290, a specific p38 inhibitor, abolished the chemotactic effect of conditioned media, whereas it did not modify the chemotactic effect induced by calcium addition (Fig 4A). These results suggest that conditioned media induced migration is p38 dependent. However, p38 activity is not essential for calcium stimulated chemotaxis.

### 3.5 Conditioned media induced chemotactic effect is calcium dependent

**Fig 5:**
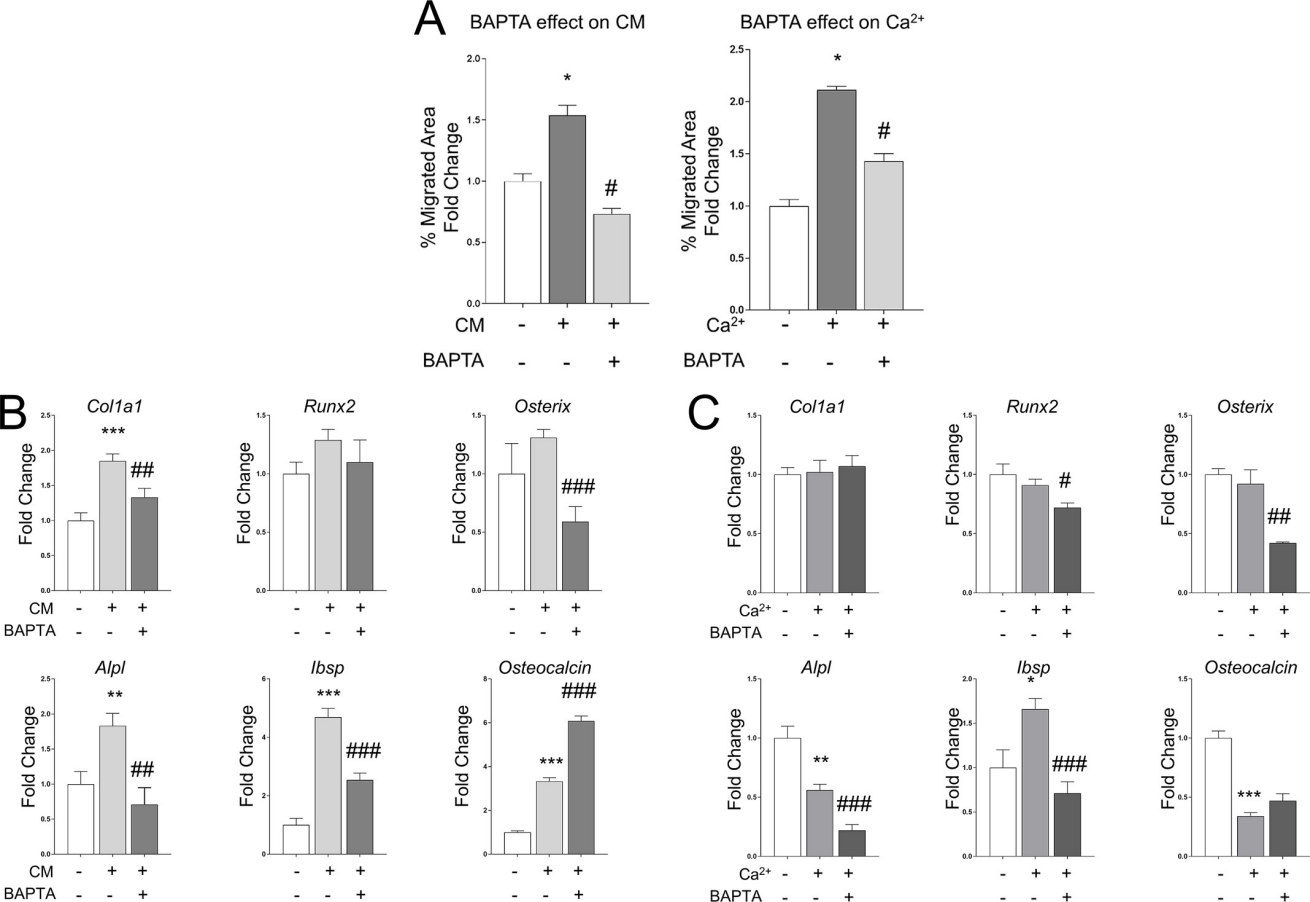
(A) A wound healing assay was used to test the role of cytosolic calcium in the calcium and conditioned media induced chemotaxis. Cells were treated with BAPTA-AM (10 μM), the cell monolayer was scratched, and cell migration was quantified after 24 hours (B) Primary calvarial cells were treated with BAPTA-AM (10 μM), and cultured with conditioned media. After 48 hours, the expression of key osteogenic genes was quantified by qPCR. (C). Calvarial cells treated with BAPTA-AM (10 μM) were cultured with 5mM CaCl_2_ for 48 hours, and the gene expression quantified. Data is shown as mean ± SEM. Differences considered significant at *p< 0.05, **p < 0.01, and ***p < 0.001 respect to control, and # p< 0.05, ## p < 0.01, and ### p < 0.001 respect to calcium treated cells.

We have demonstrated that two different stimuli, extracellular calcium and conditioned media, induce an equivalent chemotactic cell response. In search of molecular mechanisms for this common chemotactic effect, we investigated the role of cytosolic calcium. We found that BAPTA-AM treatment (10 μM), a cytosolic calcium chelator, down-regulated significantly the chemotactic effect promoted not only by extracellular calcium, but also by conditioned media (Fig 5A).We also found that BAPTA-AM treatment also produced a significant decrease in the *Osterix*, *Alpl* and *Ibsp* mRNA expression induced by both stimuli (Fig 5B and 5C). In contrast, this inhibition in the elevation of cytosolic calcium by BAPTA-AM up-regulated the *Osteocalcin* mRNA expression in conditioned media treated cells. These results suggest that the raising of cytosolic calcium concentrations is an essential event to promote the conditioned media induced chemotaxis.

### 3.6 Calcium induce the chemotactic cell migration in parallel to transiently attenuate the osteogenic gene expression

**Fig 6:**
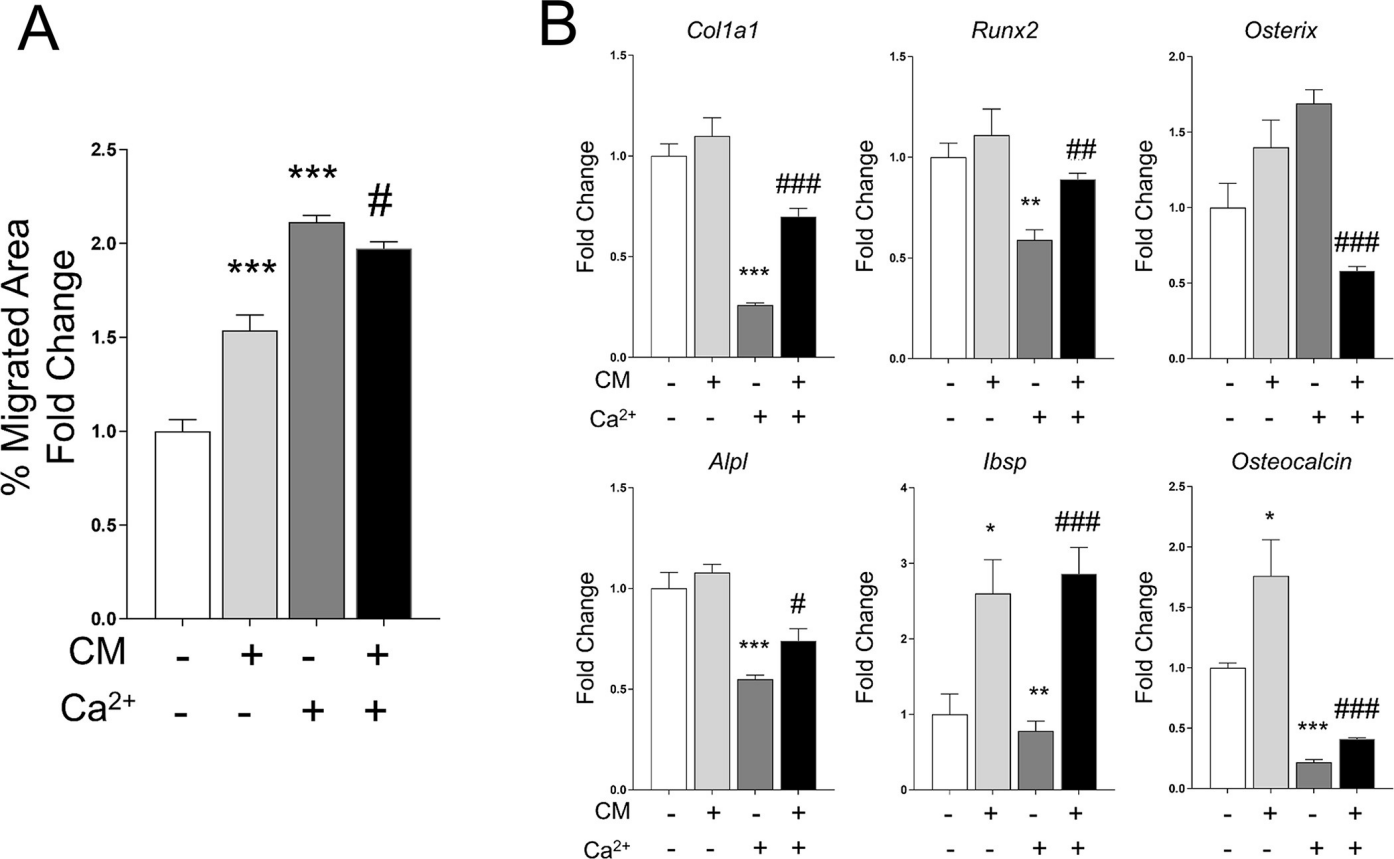
(A) The chemotactic response of primary calvarial cells to 5 mM CaCl_2_ alone or in combination with conditioned media was assessed by using a wound healing assay. After 24 hours, the number of migrated cells was quantified. (B) A parallel experiment was performed to assess the osteogenic marker expression, after 48 hours. Data is shown as mean ± SEM. Differences considered significant at *p< 0.05, **p < 0.01, and ***p < 0.001 respect to control, and # p< 0.05, ## p < 0.01, and ### p < 0.001 respect to calcium treated cells.

**Fig 7:**
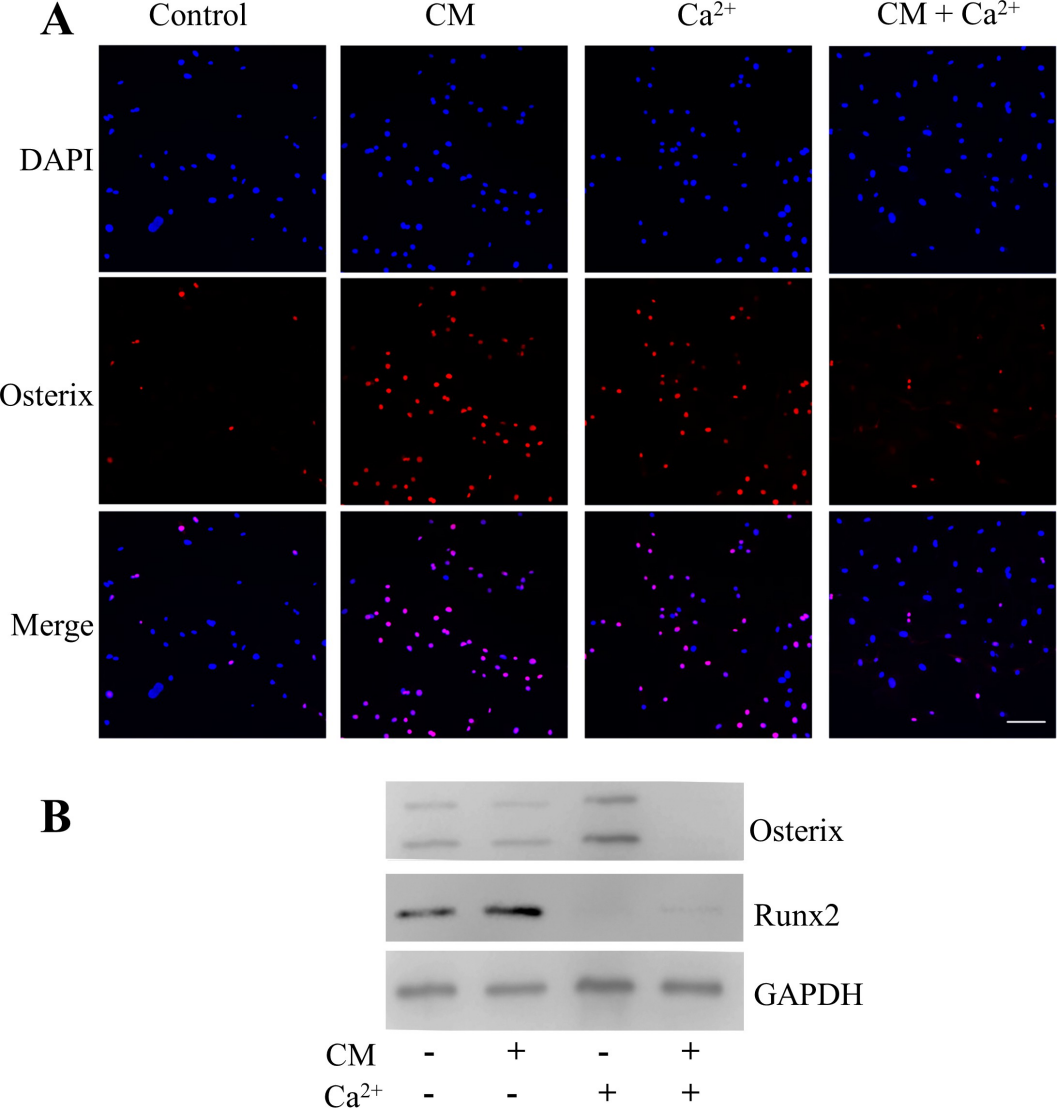
(A) Immunofluorescence staining of Osterix (red) and nuclei (blue). Calvarial cells were treated with conditioned media or 5mM calcium alone, or in combination for 48 hours. Scale bar 20X. (B) Western blot analyses of cell lysates was performed to detect the Osterix and Runx2 protein levels after the treatment with the different conditions for 48 hours. GAPDH was used as internal control.

We also evaluate the effect of both conditions acting independently or in combination. Using a wound healing assay, we observed a consistent chemoattractant effect induced by calcium and conditioned media. However, combination of both stimuli did not induce additive effects (Fig 6A).We also evaluated the effect produced on the osteogenic marker expression. (Fig 6B) Our results show a significant down-regulation in *Col1a1*, *Alpl*, *Runx2*, *Ibsp* and *Osteocalcin* expression induced by calcium alone compared to conditioned media. Interestingly, combination of both stimuli partially reverted the down-regulation induced by Ca^2+^ in the *Col1a1*, *Alpl*, *Runx2*, *Ibsp* and *Osteocalcin* gene expression. In agreement with these results, the Runx2 and Osterix protein levels displayed a similar pattern to that seen in the mRNA expression when analyzed by western blot, or by immunofluoresence with Osterix antibody (Fig 7). These results suggest that conditioned media may promote the chemotactic cell migration, and simultaneously produce an early osteogenic commitment at 48 hours. In contrast, calcium may induce the cell recruitment by delaying this early osteoblastic cell commitment.

## Discussion

In the present study, we demonstrate that extracellular calcium and conditioned media promote a similar chemotactic effect on primary calvarial cells. Consistently, an *ex vivo* calvarial explant culture model also showed that both stimuli have a similar effect on cell migration and bone formation. A remarkable finding in our study was that the μCT evaluation showed an equivalent *in vivo* osteogenic potential of the calcium-containing scaffolds, scaffolds seeded with MSC’s and the conditioned media-containing scaffolds.

The regenerative potential of MSC’s has been associated to their ability to differentiate into bone forming osteoblasts. However, recent evidence suggest that such bone regeneration potential is also potentiated by the paracrine effect induced by the implanted MSC’s [6][11]. Mesenchymal stem cells produce and secrete combinations of growth factors that modulate the activity of surrounding cells, or alternatively, act by modulation of their own membrane receptors (autocrine effect) [39][35]. Since these paracrine bioactive factors accumulate in conditioned media during cell culture, conditioned media can promote a similar regenerative effect than transplanted cells [9].

In our previous study we demonstrated that there is an optimal range in calcium concentrations that promote the recruitment of endogenous osteoprogenitors and bone regeneration *in vivo* [23]. In the present study, we found that calcium not only promotes cell recruitment, but also up-regulates the expression of multiple cytokines in progenitor cells. In agreement with these results, Wang *et al*. reported that biphasic calcium phosphate increased the expression of several inflammatory cytokines and growth factors in macrophage cultures [40]. It is known that cell motility and response to chemotactic factors often decrease with differentiation [41][42]. Therefore, an early modulation in osteoblast differentiation is essential to promote the migration of MSC’s [23]. In this study we found that extracellular calcium up-regulates the mRNA expression of IGF1, PDGF and TGFβ1. IGF and PDGF bind to tyrosine kinase receptors and activates the MAPK pathway, which induces an antagonistic effect on BMP signaling [43]. In addition, it has also been reported that cells cultured with high calcium concentrations enhance the expression of Smad3 and decrease the *Osteocalcin*, *Ibsp* and *Col1a1* expression in MC3T3-E1 cells [44]. Besides, further evidence supports that the TGFβ/Smad3 pathway and elevated cytosolic calcium concentrations inhibit the BMP signaling, an essential pathway for osteoblast differentiation [45][46][44][47]. Moreover, calcium scaffolds increase the expression of miRNAs involved in osteogenesis [48]. Zhang *et al*. reported that calcium could also bind to BMP-2 extracellularly affecting the interaction with the BMPRIA receptor [49]. Altogether, these data suggest that calcium might induce the cell recruitment at the expense of an early anti-osteogenic effect, which delay transiently osteoblast differentiation.

**Fig 8:**
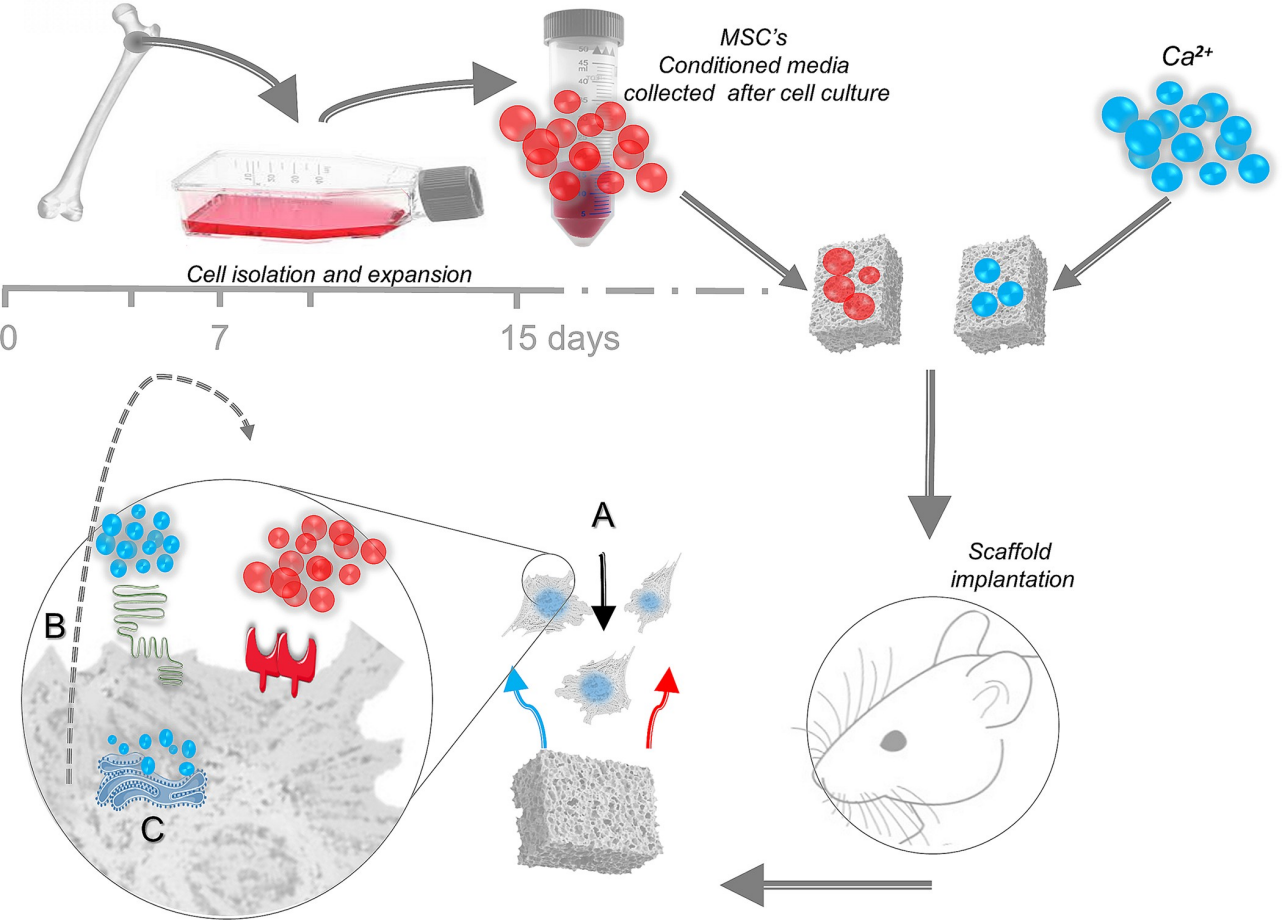
(A) Host cells infiltrate the implanted scaffold, proliferate and differentiate responding to released calcium ions. (B) Cells exposed to calcium may induce the production of a myriad of endogenous growth factors and cytokines with a paracrine and autocrine effect. (C) The raise in cytosolic calcium concentrations is required to generate both the calcium and conditioned media chemotaxis.

Moreover, calcium itself act as an extracellular signal in cells expressing the Calcium Sensing Receptor (CaSR) [50]. Indeed, CaSR activation has been demonstrated to play a prominent role in the chemotactic response of MSC’s to calcium, since its inhibition abrogates such migratory responses [24].Therefore, the beneficial effects of calcium on the endogenous cell recruitment and regeneration *in vivo* may be the result of a dual action. Initially, a direct chemotactic effect of calcium initiate the infiltration of cells into the scaffold. Over time, up-regulation of endogenous cytokines and growth factors may reinforce this cell recruitment. (Fig 8)

We also investigated the underlying molecular mechanisms that mediate the increased migration induced by calcium and conditioned media. We found that SB202190 treatment, a selective p38α/β inhibitor, completely blocked the effects of conditioned media on cell motility. However, the SB202190 treatment did not affect the calcium effects on calvarial cell migration. Consistently, the inhibition of p38 in RAW 264.7 cells, a macrophage precursor cell line, do not have significant effect on calcium-induced migration, suggesting that this pathway is not required for the chemotactic response in osteoclats [51].

Since PDGF, IGF1 and TGFβ1 [52][53][54] trigger a transient rise in the cytosolic calcium concentration, we further analyzed whether this intracellular event could link, at least partially, both calcium and conditioned media chemotactic stimuli. We found that BAPTA-AM treatment significantly decreased the chemotactic effect of both the calcium and conditioned media on calvarial cells. Furthermore, a down-regulation on key osteogenic key markers was produced by the cytosolic calcium chelation. In agreement with these results, it has been previously reported that myoblast and vascular smooth muscle cell migration also depends on the elevation of cytosolic calcium concentrations, when stimulated by PDGF, FGF, HGF or IGF1 [55][56]. Altogether, we suggest that the rise in cytosolic calcium may integrate the different signaling pathways triggered by conditioned media and extracellular calcium.

## Conclusion

We demonstrated that cell-free scaffolds containing Ca^2+^ mimic the chemotactic effect of conditioned media. In addition, calcium also induce a bone regenerative effect similar to MSC’s *in vivo*. Calcium itself initiates cell infiltration into the scaffold, and over time a paracrine effect might be produced by up-regulating the expression of multiple endogenous cytokines. Furthermore, the p38 activity is differentially required by extracellular calcium and conditioned media to promote the cell recruitment. However, the elevation of cytosolic calcium is an essential event for both stimuli to induce the chemotactic cell response. The calcium-driven *in situ* bone regeneration approach could eliminate the requirement for *ex vivo* cell isolation and manipulation, and also the need of recombinant growth factors.

## Supporting information

S1 Table Primer sequences for qPCR analysis. (XLSX)
